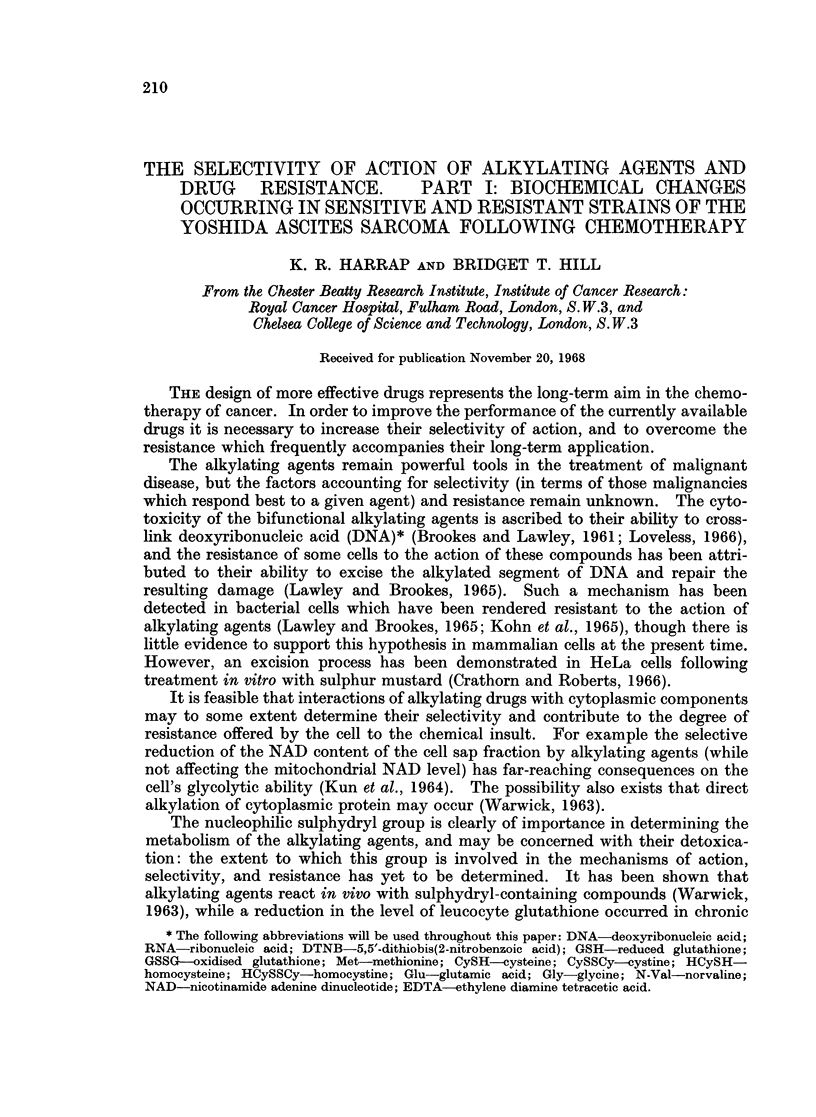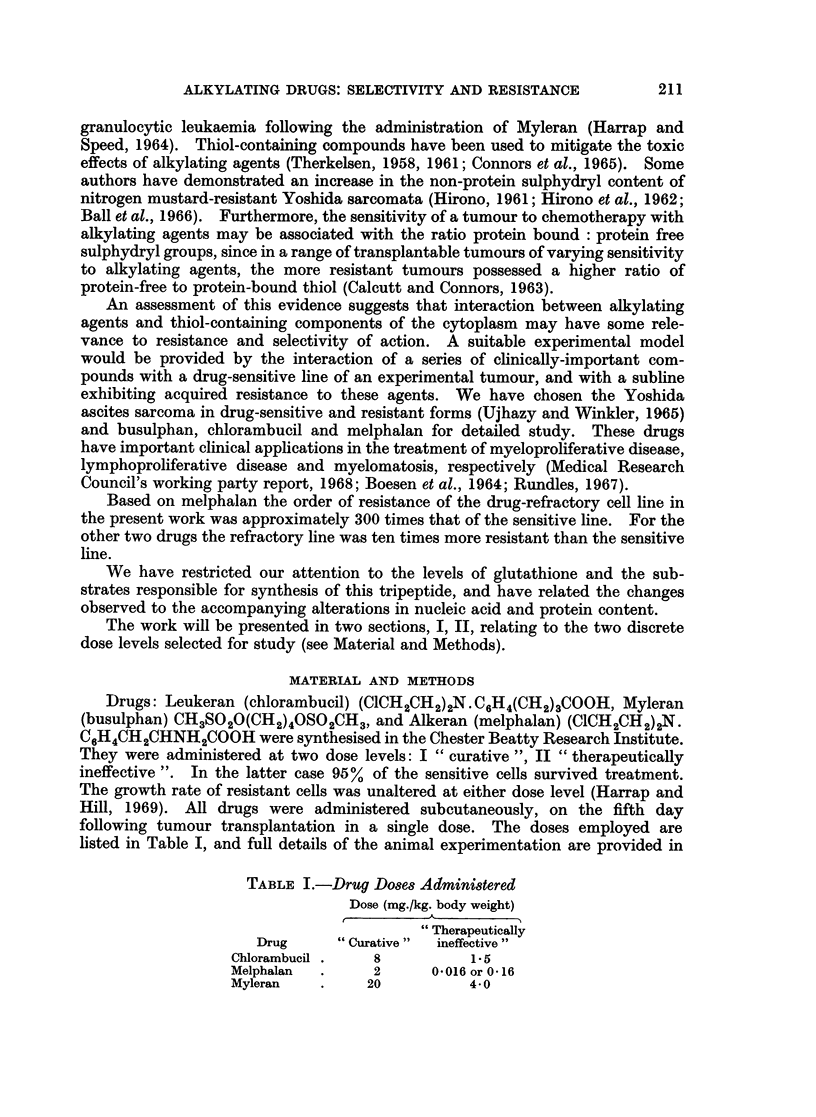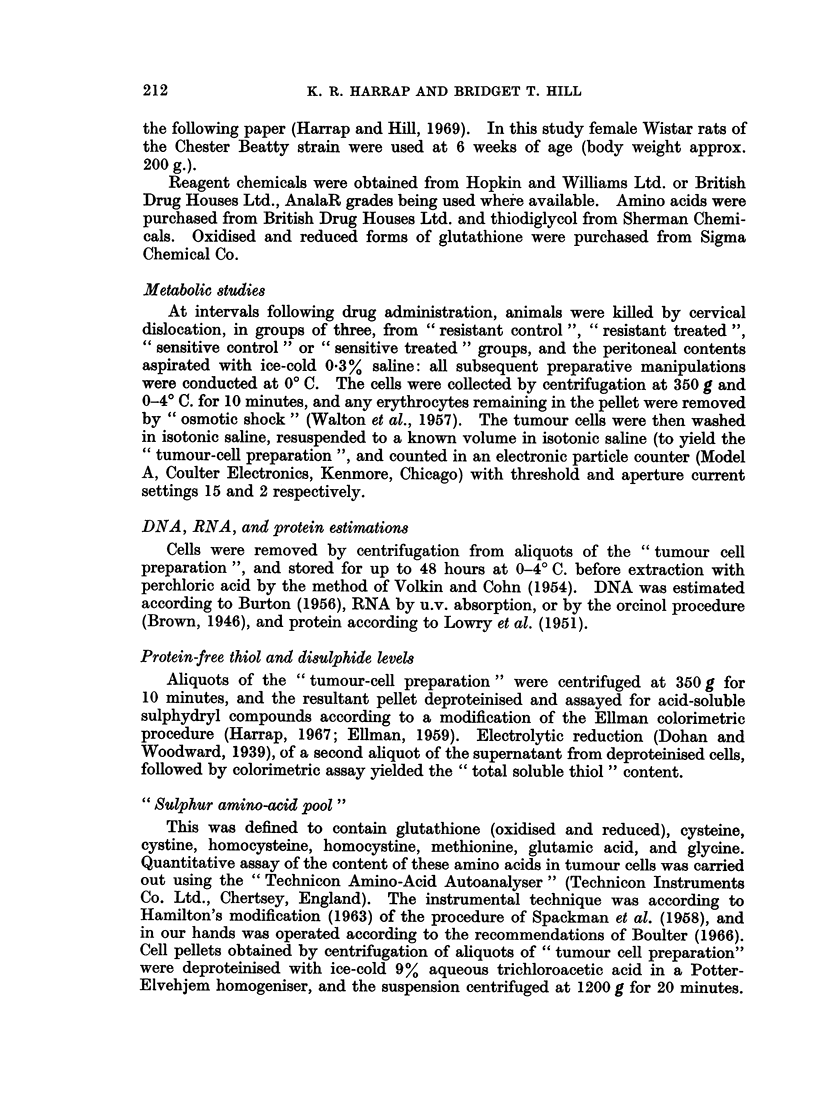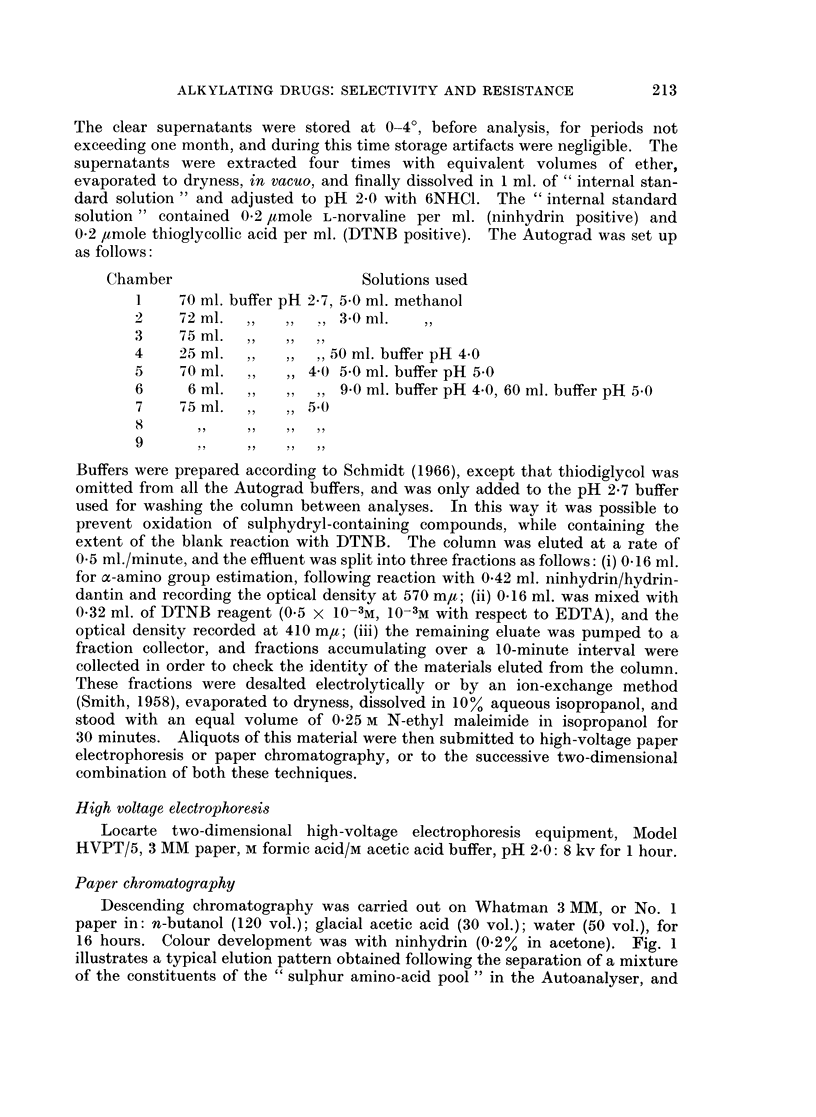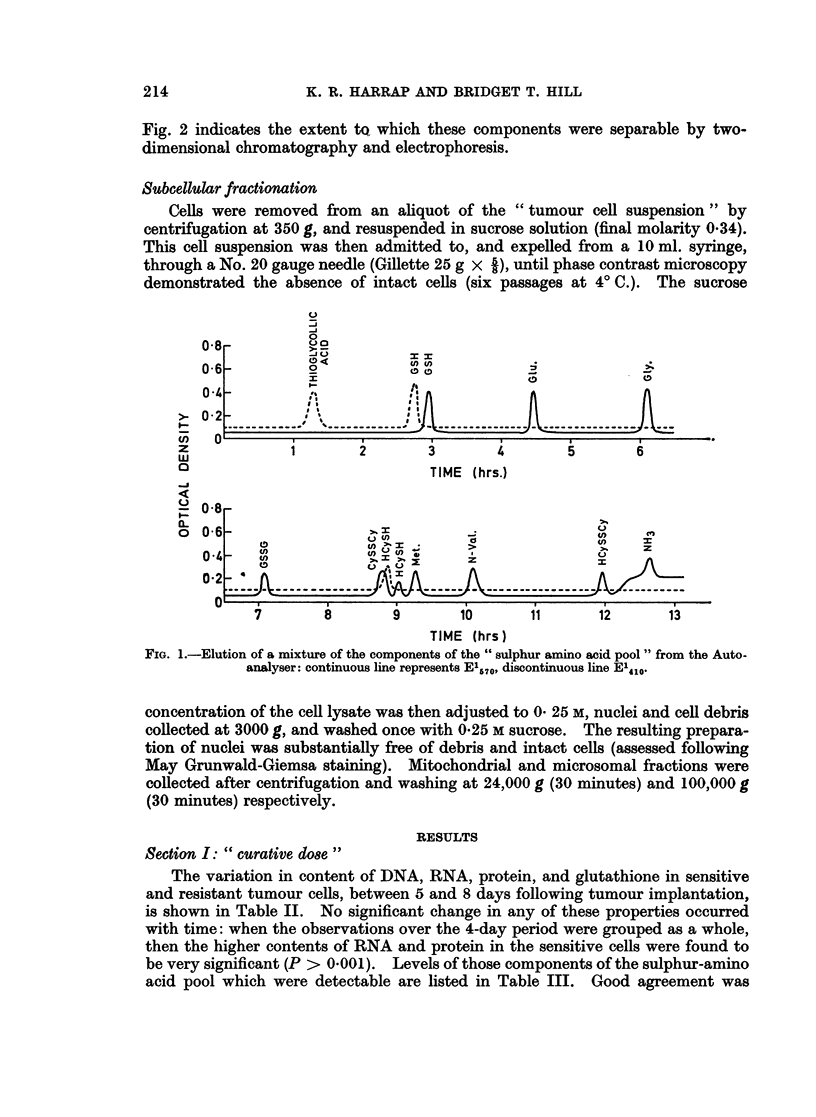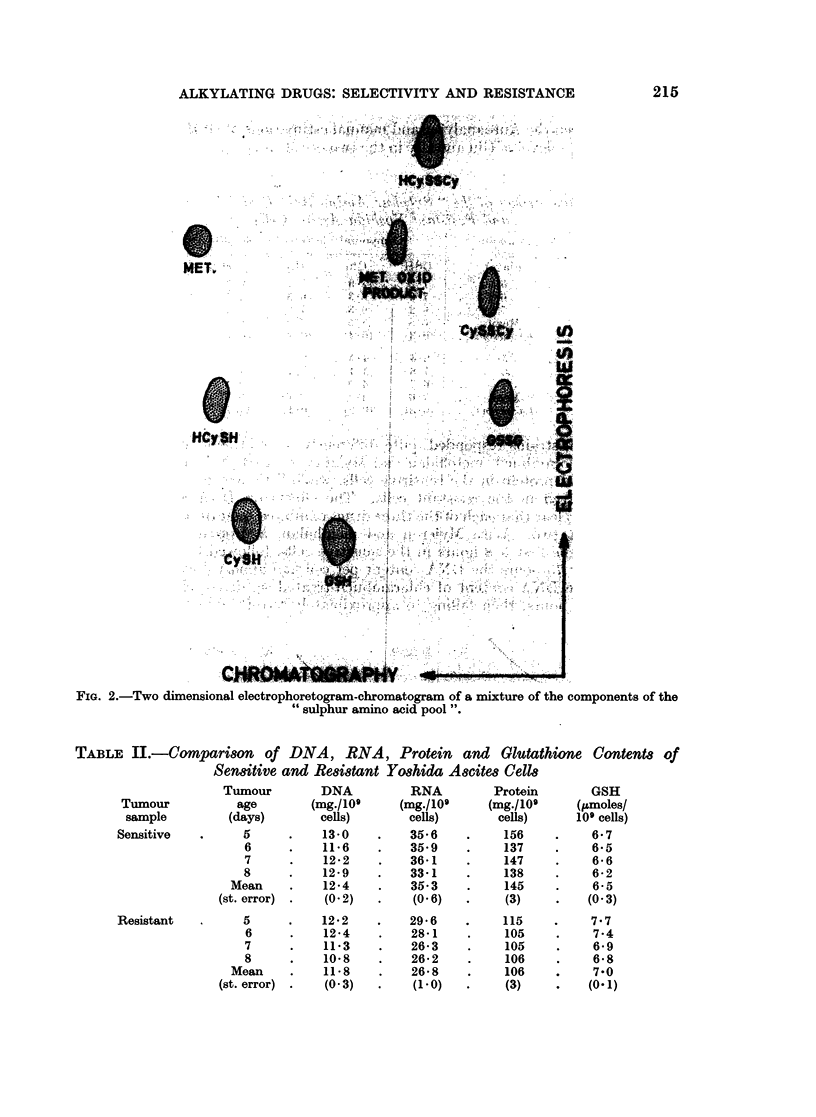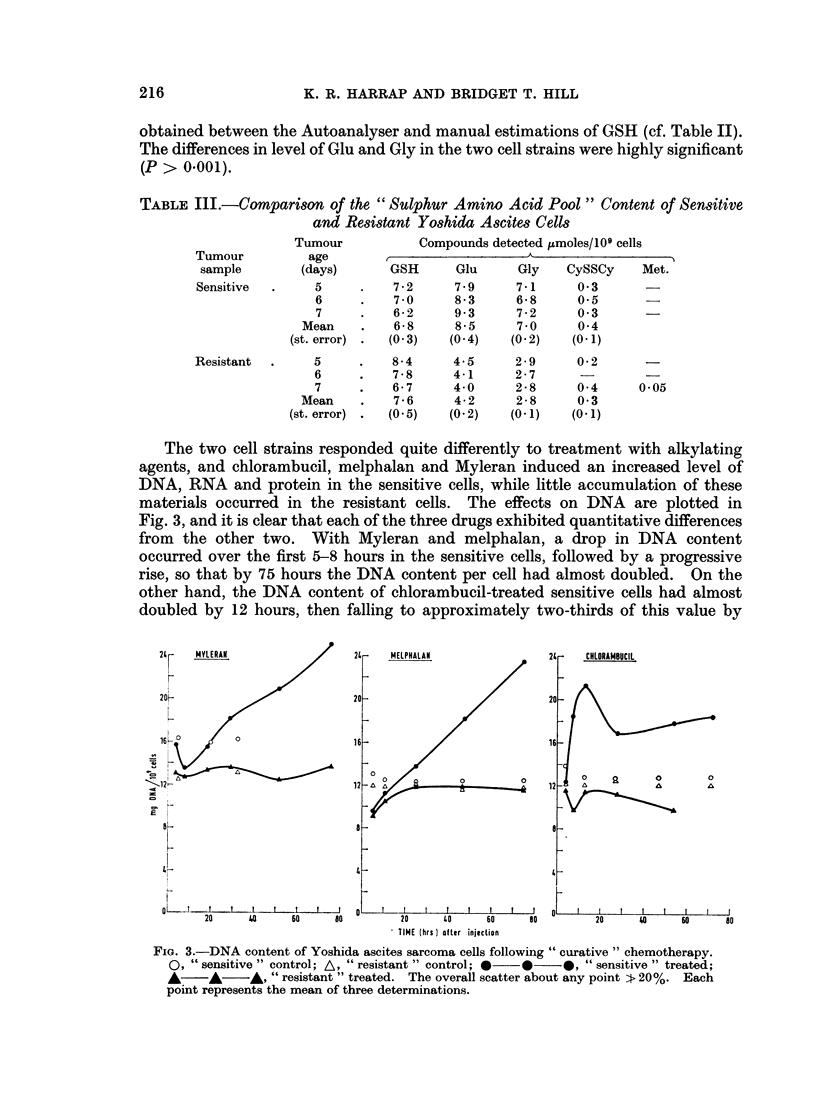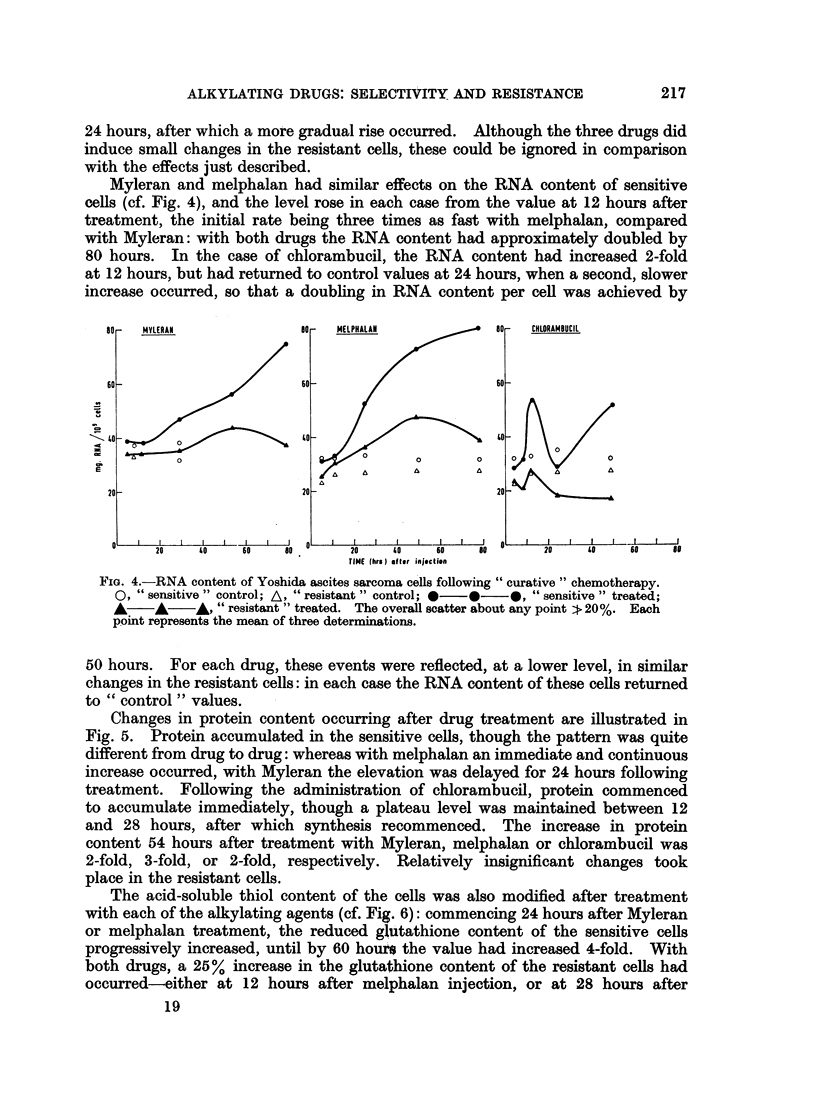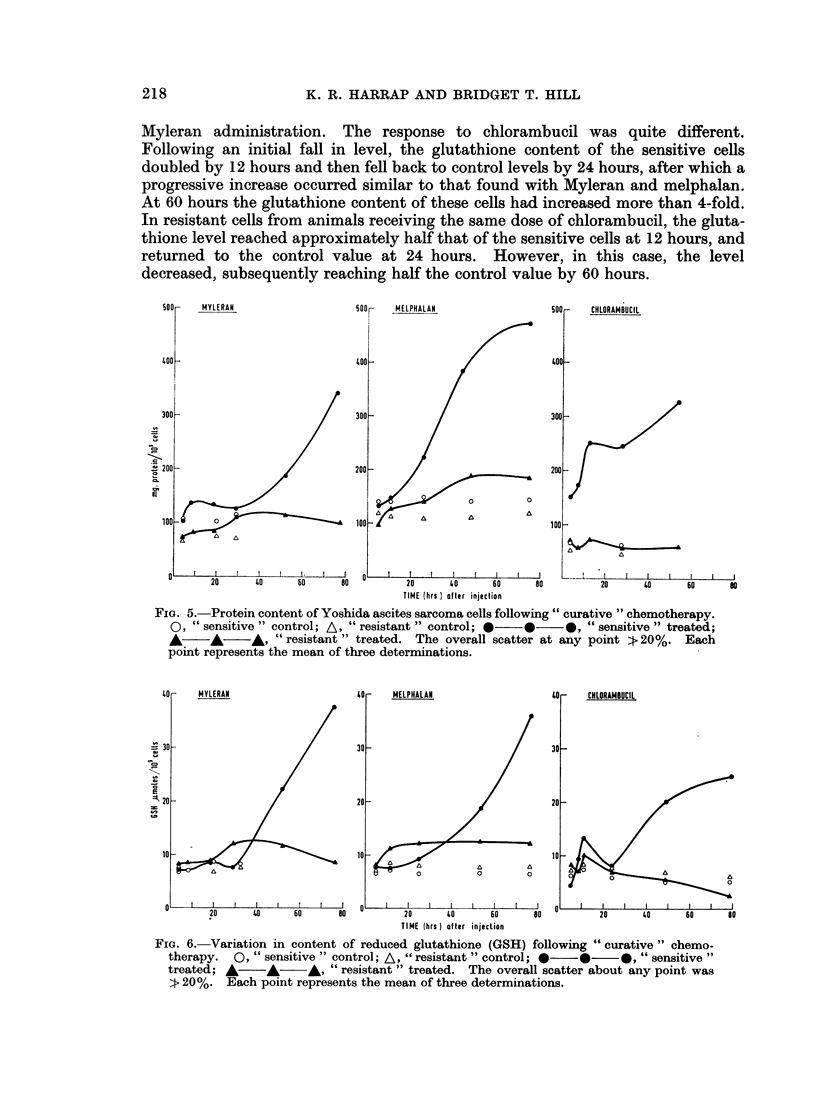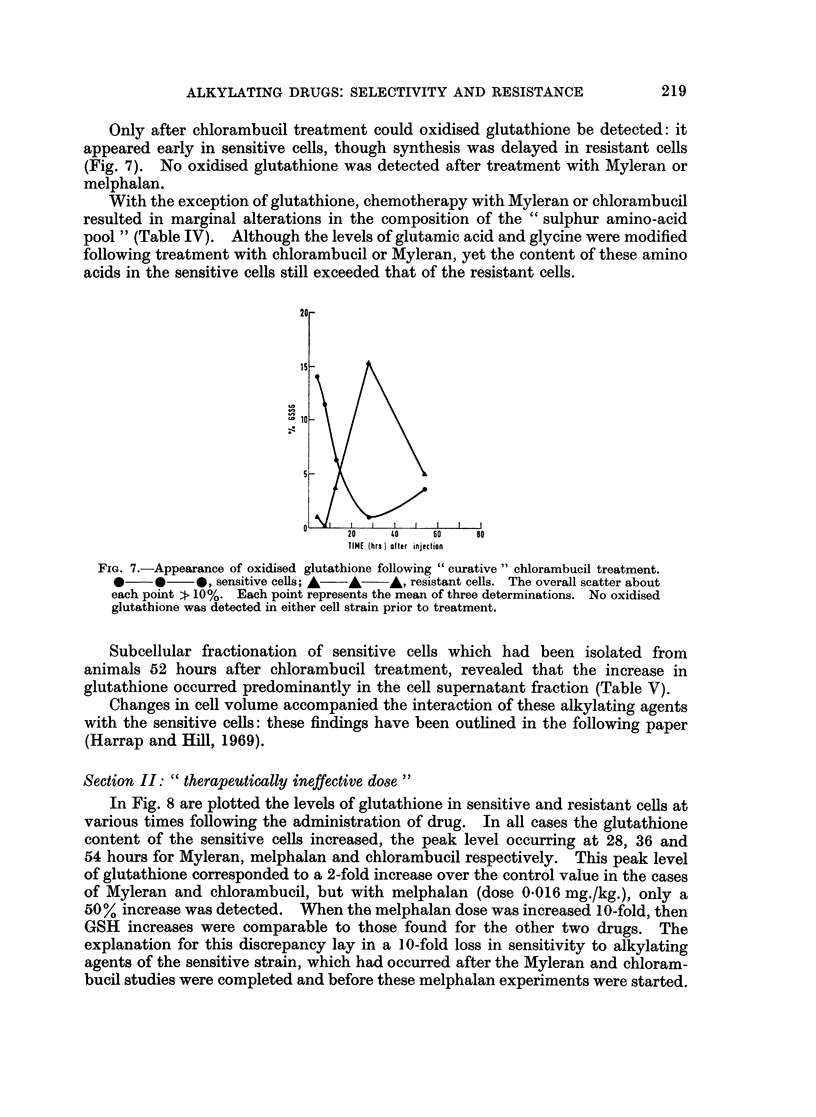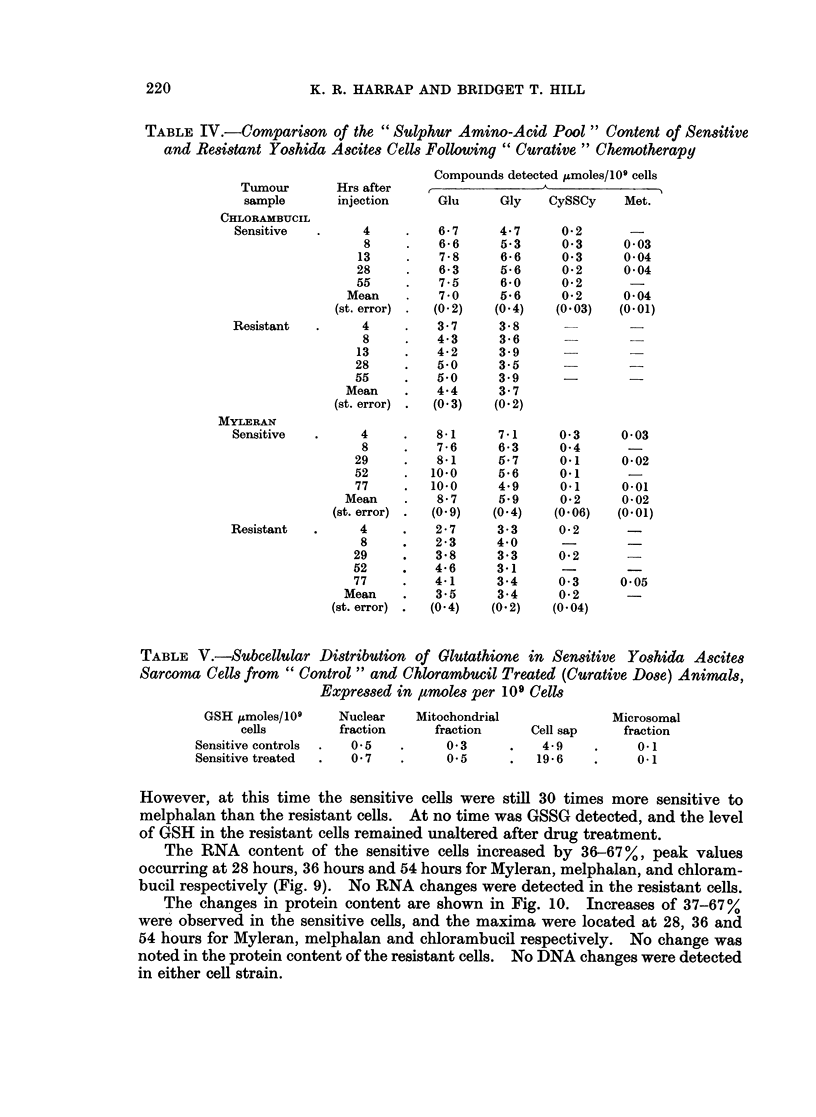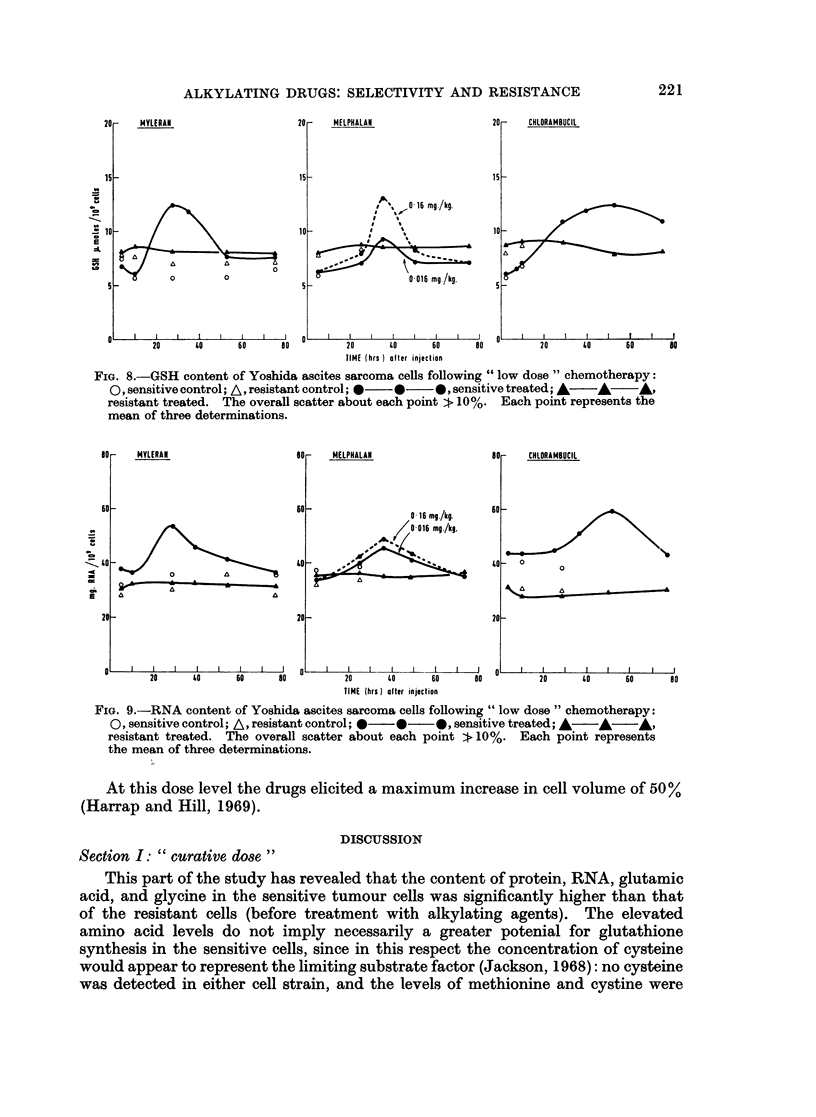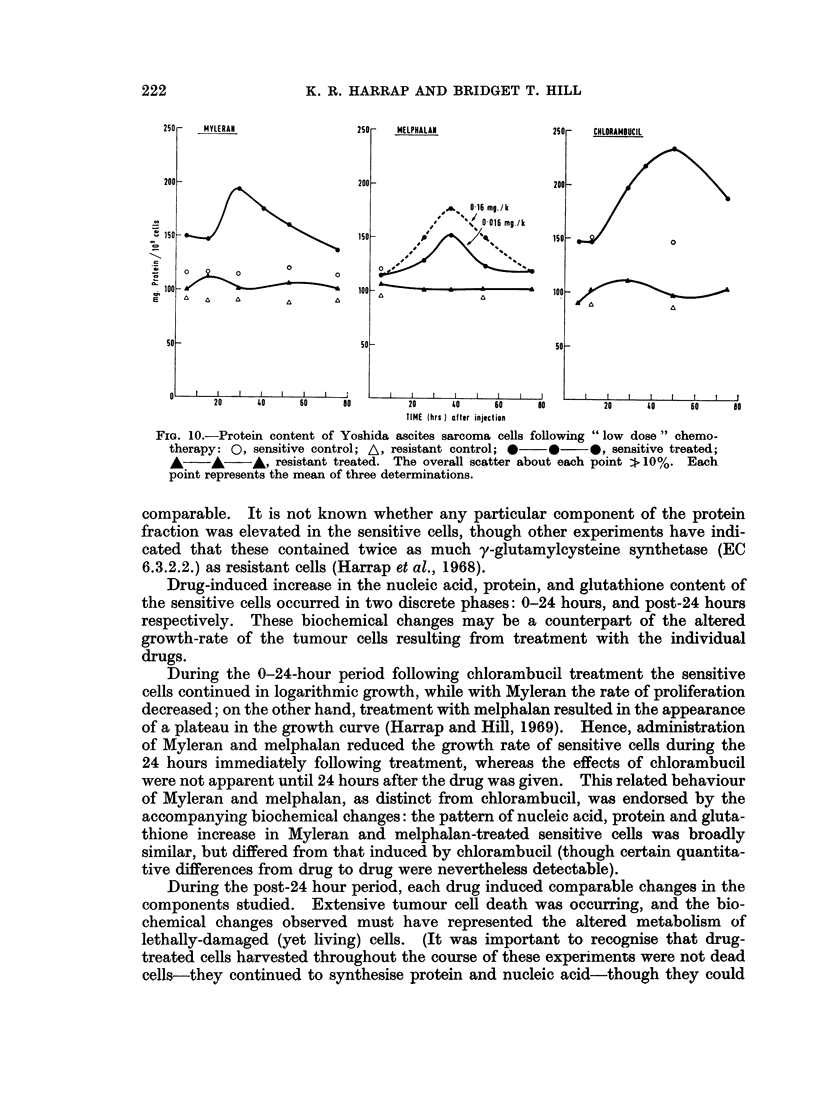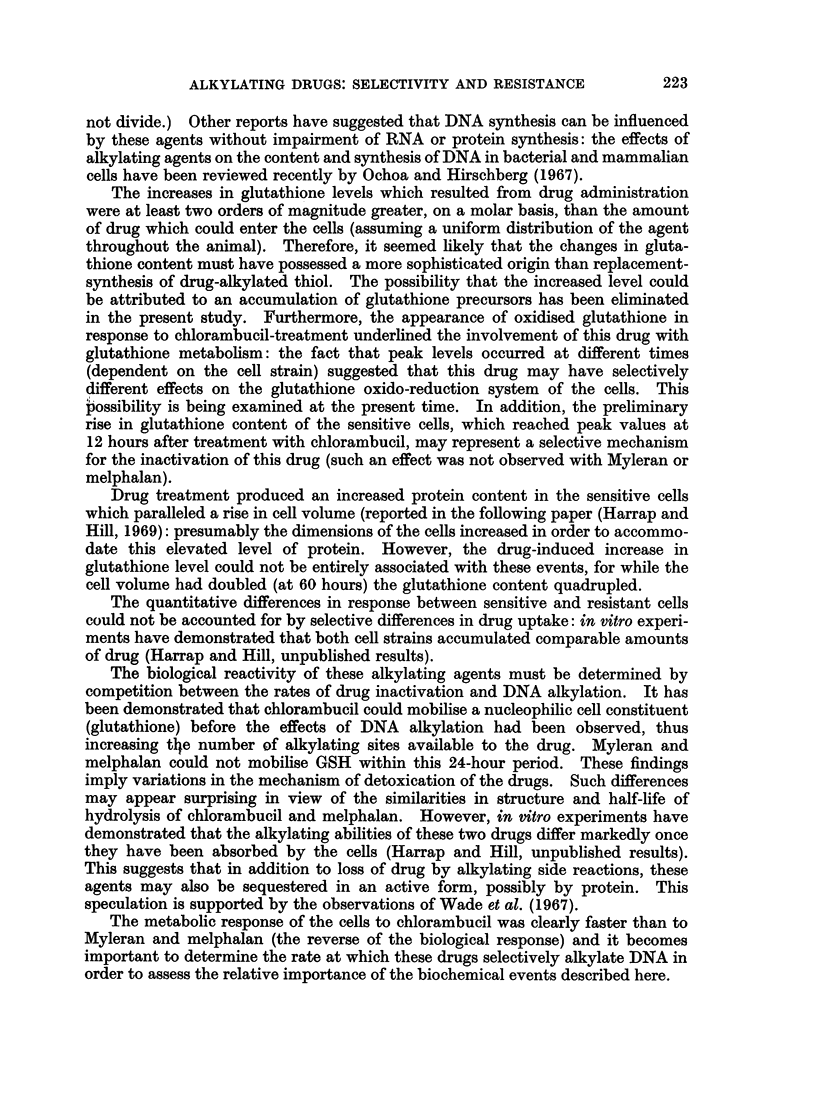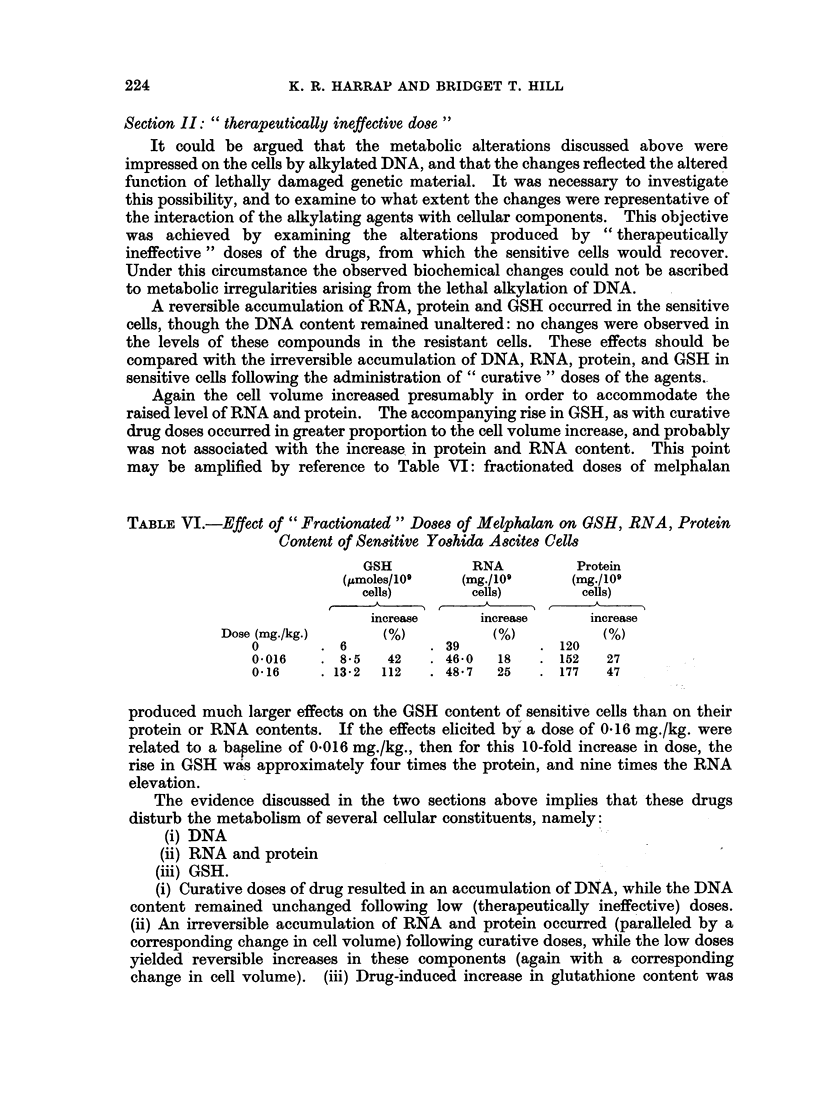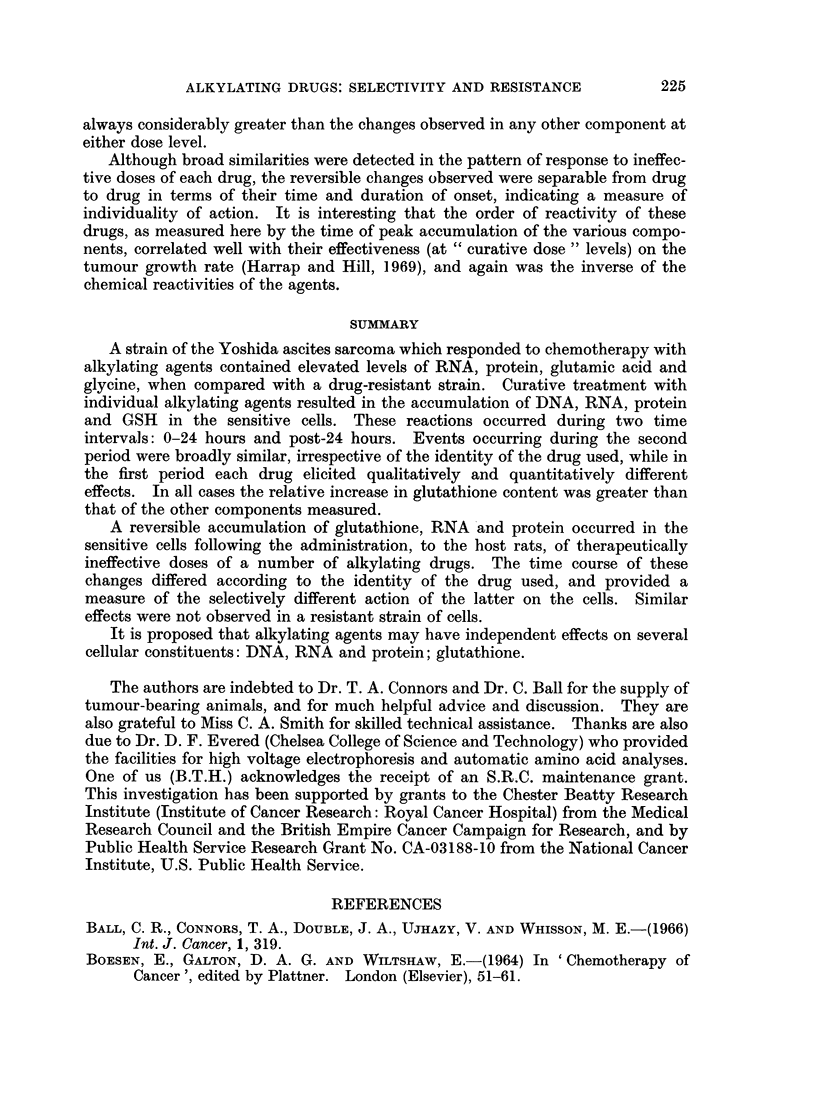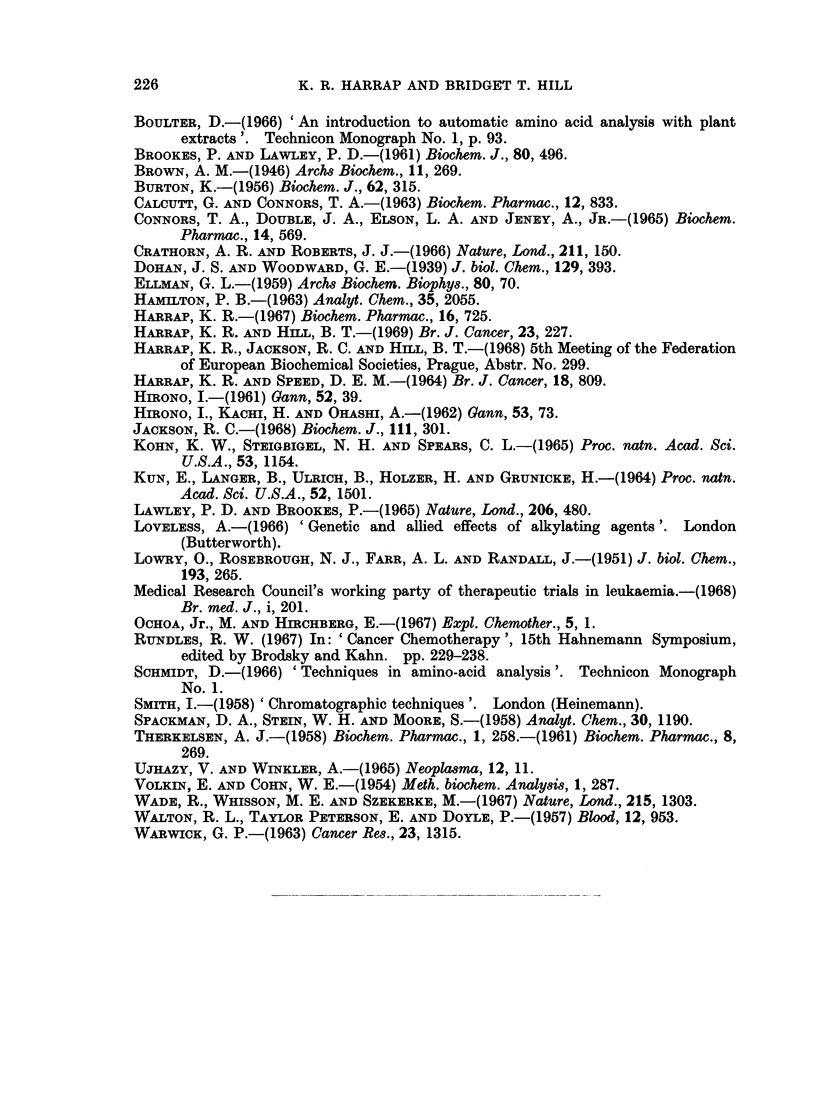# The selectivity of action of alkylating agents and drug resistance. I. Biochemical changes occurring in sensitive and resistant strains of the Yoshida ascites sarcoma following chemotherapy.

**DOI:** 10.1038/bjc.1969.30

**Published:** 1969-03

**Authors:** K. R. Harrap, B. T. Hill


### THE SELECTIVITY OF ACTION OF ALKYLATING AGENTS AND DRUG RESISTANCE. PART I: BIOCHEMICAL CHANGES OCCURRING IN SENSITIVE AND RESISTANT STRAINS OF THE YOSHIDA ASCITES SARCOMA FOLLOWING CHEMOTHERAPY

K. R. HARRAP AND BRIDGET T. HILL

*From the Chester Beatty Research Institute, Institute of Cancer Research: Royal Cancer Hospital, Fulham Road, London, S.W.3, and Chelsea College of Science and Technology, London, S.W.3*

Received for publication November 20, 1968

THE design of more effective drugs represents the long-term aim in the chemotherapy of cancer. In order to improve the performance of the currently available drugs it is necessary to increase their selectivity of action, and to overcome the resistance which frequently accompanies their long-term application.

The alkylating agents remain powerful tools in the treatment of malignant disease, but the factors accounting for selectivity (in terms of those malignancies which respond best to a given agent) and resistance remain unknown. The cytotoxicity of the bifunctional alkylating agents is ascribed to their ability to cross-link deoxyribonucleic acid (DNA)* (Brookes and Lawley, 1961; Loveless, 1966), and the resistance of some cells to the action of these compounds has been attributed to their ability to excise the alkylated segment of DNA and repair the resulting damage (Lawley and Brookes, 1965). Such a mechanism has been detected in bacterial cells which have been rendered resistant to the action of alkylating agents (Lawley and Brookes, 1965; Kohn *et al.*, 1965), though there is little evidence to support this hypothesis in mammalian cells at the present time. However, an excision process has been demonstrated in HeLa cells following treatment *in vitro* with sulphur mustard (Crathorn and Roberts, 1966).

It is feasible that interactions of alkylating drugs with cytoplasmic components may to some extent determine their selectivity and contribute to the degree of resistance offered by the cell to the chemical insult. For example the selective reduction of the NAD content of the cell sap fraction by alkylating agents (while not affecting the mitochondrial NAD level) has far-reaching consequences on the cell's glycolytic ability (Kun *et al.*, 1964). The possibility also exists that direct alkylation of cytoplasmic protein may occur (Warwick, 1963).

The nucleophilic sulphydryl group is clearly of importance in determining the metabolism of the alkylating agents, and may be concerned with their detoxication: the extent to which this group is involved in the mechanisms of action, selectivity, and resistance has yet to be determined. It has been shown that alkylating agents react *in vivo* with sulphydryl-containing compounds (Warwick, 1963), while a reduction in the level of leucocyte glutathione occurred in chronic

* The following abbreviations will be used throughout this paper: DNA—deoxyribonucleic acid; RNA—ribonucleic acid; DTNB—5,5′-dithiobis(2-nitrobenzoic acid); GSH—reduced glutathione; GSSG—oxidised glutathione; Met—methionine; CySH—cysteine; CySSCy—cystine; HCySH—homocysteine; HCySSCy—homocystine; Glu—glutamic acid; Gly—glycine; N-Val—norvaline; NAD—nicotinamide adenine dinucleotide; EDTA—ethylene diamine tetracetic acid.

granulocytic leukaemia following the administration of Myleran (Harrap and Speed, 1964). Thiol-containing compounds have been used to mitigate the toxic effects of alkylating agents (Therkelsen, 1958, 1961; Connors *et al.*, 1965). Some authors have demonstrated an increase in the non-protein sulphydryl content of nitrogen mustard-resistant Yoshida sarcomata (Hirono, 1961; Hirono *et al.*, 1962; Ball *et al.*, 1966). Furthermore, the sensitivity of a tumour to chemotherapy with alkylating agents may be associated with the ratio protein bound : protein free sulphydryl groups, since in a range of transplantable tumours of varying sensitivity to alkylating agents, the more resistant tumours possessed a higher ratio of protein-free to protein-bound thiol (Calcutt and Connors, 1963).

An assessment of this evidence suggests that interaction between alkylating agents and thiol-containing components of the cytoplasm may have some relevance to resistance and selectivity of action. A suitable experimental model would be provided by the interaction of a series of clinically-important compounds with a drug-sensitive line of an experimental tumour, and with a subline exhibiting acquired resistance to these agents. We have chosen the Yoshida ascites sarcoma in drug-sensitive and resistant forms (Ujhazy and Winkler, 1965) and busulphan, chlorambucil and melphalan for detailed study. These drugs have important clinical applications in the treatment of myeloproliferative disease, lymphoproliferative disease and myelomatosis, respectively (Medical Research Council's working party report, 1968; Boesen *et al.*, 1964; Rundles, 1967).

Based on melphalan the order of resistance of the drug-refractory cell line in the present work was approximately 300 times that of the sensitive line. For the other two drugs the refractory line was ten times more resistant than the sensitive line.

We have restricted our attention to the levels of glutathione and the substrates responsible for synthesis of this tripeptide, and have related the changes observed to the accompanying alterations in nucleic acid and protein content.

The work will be presented in two sections, I, II, relating to the two discrete dose levels selected for study (see Material and Methods).

### MATERIAL AND METHODS

Drugs: Leukeran (chlorambucil) $(ClCH_2CH_2)_2N.C_6H_4(CH_2)_3COOH$, Myleran (busulphan) $CH_3SO_2O(CH_2)_4OSO_2CH_3$, and Alkeran (melphalan) $(ClCH_2CH_2)_2N.C_6H_4CH_2CHNH_2COOH$ were synthesised in the Chester Beatty Research Institute. They were administered at two dose levels: I " curative ", II " therapeutically ineffective ". In the latter case 95% of the sensitive cells survived treatment. The growth rate of resistant cells was unaltered at either dose level (Harrap and Hill, 1969). All drugs were administered subcutaneously, on the fifth day following tumour transplantation in a single dose. The doses employed are listed in Table I, and full details of the animal experimentation are provided in

TABLE I.—*Drug Doses Administered*

| Drug | Dose (mg./kg. body weight) " Curative " | " Therapeutically ineffective " |
|---|---|---|
| Chlorambucil | 8 | 1·5 |
| Melphalan | 2 | 0·016 or 0·16 |
| Myleran | 20 | 4·0 |

the following paper (Harrap and Hill, 1969). In this study female Wistar rats of the Chester Beatty strain were used at 6 weeks of age (body weight approx. 200 g.).

Reagent chemicals were obtained from Hopkin and Williams Ltd. or British Drug Houses Ltd., AnalaR grades being used where available. Amino acids were purchased from British Drug Houses Ltd. and thiodiglycol from Sherman Chemicals. Oxidised and reduced forms of glutathione were purchased from Sigma Chemical Co.

*Metabolic studies*

At intervals following drug administration, animals were killed by cervical dislocation, in groups of three, from " resistant control ", " resistant treated ", " sensitive control " or " sensitive treated " groups, and the peritoneal contents aspirated with ice-cold 0·3% saline: all subsequent preparative manipulations were conducted at 0° C. The cells were collected by centrifugation at 350 *g* and 0–4° C. for 10 minutes, and any erythrocytes remaining in the pellet were removed by " osmotic shock " (Walton *et al.*, 1957). The tumour cells were then washed in isotonic saline, resuspended to a known volume in isotonic saline (to yield the " tumour-cell preparation ", and counted in an electronic particle counter (Model A, Coulter Electronics, Kenmore, Chicago) with threshold and aperture current settings 15 and 2 respectively.

*DNA, RNA, and protein estimations*

Cells were removed by centrifugation from aliquots of the " tumour cell preparation ", and stored for up to 48 hours at 0–4° C. before extraction with perchloric acid by the method of Volkin and Cohn (1954). DNA was estimated according to Burton (1956), RNA by u.v. absorption, or by the orcinol procedure (Brown, 1946), and protein according to Lowry *et al.* (1951).

*Protein-free thiol and disulphide levels*

Aliquots of the " tumour-cell preparation " were centrifuged at 350 *g* for 10 minutes, and the resultant pellet deproteinised and assayed for acid-soluble sulphydryl compounds according to a modification of the Ellman colorimetric procedure (Harrap, 1967; Ellman, 1959). Electrolytic reduction (Dohan and Woodward, 1939), of a second aliquot of the supernatant from deproteinised cells, followed by colorimetric assay yielded the " total soluble thiol " content.

*" Sulphur amino-acid pool "*

This was defined to contain glutathione (oxidised and reduced), cysteine, cystine, homocysteine, homocystine, methionine, glutamic acid, and glycine. Quantitative assay of the content of these amino acids in tumour cells was carried out using the " Technicon Amino-Acid Autoanalyser " (Technicon Instruments Co. Ltd., Chertsey, England). The instrumental technique was according to Hamilton's modification (1963) of the procedure of Spackman *et al.* (1958), and in our hands was operated according to the recommendations of Boulter (1966). Cell pellets obtained by centrifugation of aliquots of " tumour cell preparation" were deproteinised with ice-cold 9% aqueous trichloroacetic acid in a Potter-Elvehjem homogeniser, and the suspension centrifuged at 1200 *g* for 20 minutes.

The clear supernatants were stored at 0–4°, before analysis, for periods not exceeding one month, and during this time storage artifacts were negligible. The supernatants were extracted four times with equivalent volumes of ether, evaporated to dryness, *in vacuo*, and finally dissolved in 1 ml. of " internal standard solution " and adjusted to pH 2·0 with 6NHCl. The " internal standard solution " contained 0·2 $\mu$mole L-norvaline per ml. (ninhydrin positive) and 0·2 $\mu$mole thioglycollic acid per ml. (DTNB positive). The Autograd was set up as follows:

| Chamber | Solutions used | | | | |
|---|---|---|---|---|---|
| 1 | 70 ml. | buffer | pH | 2·7, | 5·0 ml. methanol |
| 2 | 72 ml. | ,, | ,, | ,, | 3·0 ml. ,, |
| 3 | 75 ml. | ,, | ,, | ,, | |
| 4 | 25 ml. | ,, | ,, | ,, | 50 ml. buffer pH 4·0 |
| 5 | 70 ml. | ,, | ,, | 4·0 | 5·0 ml. buffer pH 5·0 |
| 6 | 6 ml. | ,, | ,, | ,, | 9·0 ml. buffer pH 4·0, 60 ml. buffer pH 5·0 |
| 7 | 75 ml. | ,, | ,, | 5·0 | |
| 8 | ,, | ,, | ,, | ,, | |
| 9 | ,, | ,, | ,, | ,, | |

Buffers were prepared according to Schmidt (1966), except that thiodiglycol was omitted from all the Autograd buffers, and was only added to the pH 2·7 buffer used for washing the column between analyses. In this way it was possible to prevent oxidation of sulphydryl-containing compounds, while containing the extent of the blank reaction with DTNB. The column was eluted at a rate of 0·5 ml./minute, and the effluent was split into three fractions as follows: (i) 0·16 ml. for $\alpha$-amino group estimation, following reaction with 0·42 ml. ninhydrin/hydrindantin and recording the optical density at 570 m$\mu$; (ii) 0·16 ml. was mixed with 0·32 ml. of DTNB reagent ($0{\cdot}5 \times 10^{-3}$M, $10^{-3}$M with respect to EDTA), and the optical density recorded at 410 m$\mu$; (iii) the remaining eluate was pumped to a fraction collector, and fractions accumulating over a 10-minute interval were collected in order to check the identity of the materials eluted from the column. These fractions were desalted electrolytically or by an ion-exchange method (Smith, 1958), evaporated to dryness, dissolved in 10% aqueous isopropanol, and stood with an equal volume of 0·25 M N-ethyl maleimide in isopropanol for 30 minutes. Aliquots of this material were then submitted to high-voltage paper electrophoresis or paper chromatography, or to the successive two-dimensional combination of both these techniques.

*High voltage electrophoresis*

Locarte two-dimensional high-voltage electrophoresis equipment, Model HVPT/5, 3 MM paper, M formic acid/M acetic acid buffer, pH 2·0: 8 kv for 1 hour.

*Paper chromatography*

Descending chromatography was carried out on Whatman 3 MM, or No. 1 paper in: *n*-butanol (120 vol.); glacial acetic acid (30 vol.); water (50 vol.), for 16 hours. Colour development was with ninhydrin (0·2% in acetone). Fig. 1 illustrates a typical elution pattern obtained following the separation of a mixture of the constituents of the " sulphur amino-acid pool " in the Autoanalyser, and

Fig. 2 indicates the extent to which these components were separable by two-dimensional chromatography and electrophoresis.

*Subcellular fractionation*

Cells were removed from an aliquot of the " tumour cell suspension " by centrifugation at 350 *g*, and resuspended in sucrose solution (final molarity 0·34). This cell suspension was then admitted to, and expelled from a 10 ml. syringe, through a No. 20 gauge needle (Gillette 25 g × $\frac{5}{8}$), until phase contrast microscopy demonstrated the absence of intact cells (six passages at 4° C.). The sucrose

FIG. 1.—Elution of a mixture of the components of the " sulphur amino acid pool " from the Autoanalyser: continuous line represents $E^{1}_{570}$, discontinuous line $E^{1}_{410}$.

concentration of the cell lysate was then adjusted to 0· 25 M, nuclei and cell debris collected at 3000 *g*, and washed once with 0·25 M sucrose. The resulting preparation of nuclei was substantially free of debris and intact cells (assessed following May Grunwald-Giemsa staining). Mitochondrial and microsomal fractions were collected after centrifugation and washing at 24,000 *g* (30 minutes) and 100,000 *g* (30 minutes) respectively.

### RESULTS

*Section I: " curative dose "*

The variation in content of DNA, RNA, protein, and glutathione in sensitive and resistant tumour cells, between 5 and 8 days following tumour implantation, is shown in Table II. No significant change in any of these properties occurred with time: when the observations over the 4-day period were grouped as a whole, then the higher contents of RNA and protein in the sensitive cells were found to be very significant ($P > 0{\cdot}001$). Levels of those components of the sulphur-amino acid pool which were detectable are listed in Table III. Good agreement was

FIG. 2.—Two dimensional electrophoretogram-chromatogram of a mixture of the components of the " sulphur amino acid pool ".

TABLE II.—*Comparison of DNA, RNA, Protein and Glutathione Contents of Sensitive and Resistant Yoshida Ascites Cells*

| Tumour sample | Tumour age (days) | DNA (mg./10⁹ cells) | RNA (mg./10⁹ cells) | Protein (mg./10⁹ cells) | GSH (μmoles/ 10⁹ cells) |
|---|---|---|---|---|---|
| Sensitive | 5 | 13·0 | 35·6 | 156 | 6·7 |
| | 6 | 11·6 | 35·9 | 137 | 6·5 |
| | 7 | 12·2 | 36·1 | 147 | 6·6 |
| | 8 | 12·9 | 33·1 | 138 | 6·2 |
| | Mean | 12·4 | 35·3 | 145 | 6·5 |
| | (st. error) | (0·2) | (0·6) | (3) | (0·3) |
| Resistant | 5 | 12·2 | 29·6 | 115 | 7·7 |
| | 6 | 12·4 | 28·1 | 105 | 7·4 |
| | 7 | 11·3 | 26·3 | 105 | 6·9 |
| | 8 | 10·8 | 26·2 | 106 | 6·8 |
| | Mean | 11·8 | 26·8 | 106 | 7·0 |
| | (st. error) | (0·3) | (1·0) | (3) | (0·1) |

obtained between the Autoanalyser and manual estimations of GSH (cf. Table II). The differences in level of Glu and Gly in the two cell strains were highly significant ($P > 0{\cdot}001$).

TABLE III.—*Comparison of the " Sulphur Amino Acid Pool " Content of Sensitive and Resistant Yoshida Ascites Cells*

| Tumour sample | Tumour age (days) | Compounds detected µmoles/10⁹ cells GSH | Glu | Gly | CySSCy | Met. |
|---|---|---|---|---|---|---|
| Sensitive | 5 | 7·2 | 7·9 | 7·1 | 0·3 | — |
| | 6 | 7·0 | 8·3 | 6·8 | 0·5 | — |
| | 7 | 6·2 | 9·3 | 7·2 | 0·3 | — |
| | Mean | 6·8 | 8·5 | 7·0 | 0·4 | |
| | (st. error) | (0·3) | (0·4) | (0·2) | (0·1) | |
| Resistant | 5 | 8·4 | 4·5 | 2·9 | 0·2 | — |
| | 6 | 7·8 | 4·1 | 2·7 | — | — |
| | 7 | 6·7 | 4·0 | 2·8 | 0·4 | 0·05 |
| | Mean | 7·6 | 4·2 | 2·8 | 0·3 | |
| | (st. error) | (0·5) | (0·2) | (0·1) | (0·1) | |

The two cell strains responded quite differently to treatment with alkylating agents, and chlorambucil, melphalan and Myleran induced an increased level of DNA, RNA and protein in the sensitive cells, while little accumulation of these materials occurred in the resistant cells. The effects on DNA are plotted in Fig. 3, and it is clear that each of the three drugs exhibited quantitative differences from the other two. With Myleran and melphalan, a drop in DNA content occurred over the first 5–8 hours in the sensitive cells, followed by a progressive rise, so that by 75 hours the DNA content per cell had almost doubled. On the other hand, the DNA content of chlorambucil-treated sensitive cells had almost doubled by 12 hours, then falling to approximately two-thirds of this value by

FIG. 3.—DNA content of Yoshida ascites sarcoma cells following " curative " chemotherapy. ○, " sensitive " control; △, " resistant " control; ●——●——●, " sensitive " treated; ▲——▲——▲, " resistant " treated. The overall scatter about any point $\not> 20\%$. Each point represents the mean of three determinations.

24 hours, after which a more gradual rise occurred. Although the three drugs did induce small changes in the resistant cells, these could be ignored in comparison with the effects just described.

Myleran and melphalan had similar effects on the RNA content of sensitive cells (cf. Fig. 4), and the level rose in each case from the value at 12 hours after treatment, the initial rate being three times as fast with melphalan, compared with Myleran: with both drugs the RNA content had approximately doubled by 80 hours. In the case of chlorambucil, the RNA content had increased 2-fold at 12 hours, but had returned to control values at 24 hours, when a second, slower increase occurred, so that a doubling in RNA content per cell was achieved by

FIG. 4.—RNA content of Yoshida ascites sarcoma cells following " curative " chemotherapy. ○, " sensitive " control; △, " resistant " control; ●——●——●, " sensitive " treated; ▲——▲——▲, " resistant " treated. The overall scatter about any point ≯ 20%. Each point represents the mean of three determinations.

50 hours. For each drug, these events were reflected, at a lower level, in similar changes in the resistant cells: in each case the RNA content of these cells returned to " control " values.

Changes in protein content occurring after drug treatment are illustrated in Fig. 5. Protein accumulated in the sensitive cells, though the pattern was quite different from drug to drug: whereas with melphalan an immediate and continuous increase occurred, with Myleran the elevation was delayed for 24 hours following treatment. Following the administration of chlorambucil, protein commenced to accumulate immediately, though a plateau level was maintained between 12 and 28 hours, after which synthesis recommenced. The increase in protein content 54 hours after treatment with Myleran, melphalan or chlorambucil was 2-fold, 3-fold, or 2-fold, respectively. Relatively insignificant changes took place in the resistant cells.

The acid-soluble thiol content of the cells was also modified after treatment with each of the alkylating agents (cf. Fig. 6): commencing 24 hours after Myleran or melphalan treatment, the reduced glutathione content of the sensitive cells progressively increased, until by 60 hours the value had increased 4-fold. With both drugs, a 25% increase in the glutathione content of the resistant cells had occurred—either at 12 hours after melphalan injection, or at 28 hours after

Myleran administration. The response to chlorambucil was quite different. Following an initial fall in level, the glutathione content of the sensitive cells doubled by 12 hours and then fell back to control levels by 24 hours, after which a progressive increase occurred similar to that found with Myleran and melphalan. At 60 hours the glutathione content of these cells had increased more than 4-fold. In resistant cells from animals receiving the same dose of chlorambucil, the glutathione level reached approximately half that of the sensitive cells at 12 hours, and returned to the control value at 24 hours. However, in this case, the level decreased, subsequently reaching half the control value by 60 hours.

FIG. 5.—Protein content of Yoshida ascites sarcoma cells following " curative " chemotherapy. ○, " sensitive " control; △, " resistant " control; ●——●——●, " sensitive " treated; ▲——▲——▲, " resistant " treated. The overall scatter at any point $\not> 20\%$. Each point represents the mean of three determinations.

FIG. 6.—Variation in content of reduced glutathione (GSH) following " curative " chemotherapy. ○, " sensitive " control; △, " resistant " control; ●——●——●, " sensitive " treated; ▲——▲——▲, " resistant " treated. The overall scatter about any point was $\not> 20\%$. Each point represents the mean of three determinations.

Only after chlorambucil treatment could oxidised glutathione be detected: it appeared early in sensitive cells, though synthesis was delayed in resistant cells (Fig. 7). No oxidised glutathione was detected after treatment with Myleran or melphalan.

With the exception of glutathione, chemotherapy with Myleran or chlorambucil resulted in marginal alterations in the composition of the " sulphur amino-acid pool " (Table IV). Although the levels of glutamic acid and glycine were modified following treatment with chlorambucil or Myleran, yet the content of these amino acids in the sensitive cells still exceeded that of the resistant cells.

Fig. 7.—Appearance of oxidised glutathione following " curative " chlorambucil treatment. ●——●——●, sensitive cells; ▲——▲——▲, resistant cells. The overall scatter about each point ≯ 10%. Each point represents the mean of three determinations. No oxidised glutathione was detected in either cell strain prior to treatment.

Subcellular fractionation of sensitive cells which had been isolated from animals 52 hours after chlorambucil treatment, revealed that the increase in glutathione occurred predominantly in the cell supernatant fraction (Table V).

Changes in cell volume accompanied the interaction of these alkylating agents with the sensitive cells: these findings have been outlined in the following paper (Harrap and Hill, 1969).

*Section II: " therapeutically ineffective dose "*

In Fig. 8 are plotted the levels of glutathione in sensitive and resistant cells at various times following the administration of drug. In all cases the glutathione content of the sensitive cells increased, the peak level occurring at 28, 36 and 54 hours for Myleran, melphalan and chlorambucil respectively. This peak level of glutathione corresponded to a 2-fold increase over the control value in the cases of Myleran and chlorambucil, but with melphalan (dose 0·016 mg./kg.), only a 50% increase was detected. When the melphalan dose was increased 10-fold, then GSH increases were comparable to those found for the other two drugs. The explanation for this discrepancy lay in a 10-fold loss in sensitivity to alkylating agents of the sensitive strain, which had occurred after the Myleran and chlorambucil studies were completed and before these melphalan experiments were started.

TABLE IV.—*Comparison of the " Sulphur Amino-Acid Pool " Content of Sensitive and Resistant Yoshida Ascites Cells Following " Curative " Chemotherapy*

| Tumour sample | Hrs after injection | Compounds detected μmoles/10⁹ cells Glu | Gly | CySSCy | Met. |
|---|---|---|---|---|---|
| CHLORAMBUCIL | | | | | |
| Sensitive | 4 | 6·7 | 4·7 | 0·2 | — |
| | 8 | 6·6 | 5·3 | 0·3 | 0·03 |
| | 13 | 7·8 | 6·6 | 0·3 | 0·04 |
| | 28 | 6·3 | 5·6 | 0·2 | 0·04 |
| | 55 | 7·5 | 6·0 | 0·2 | — |
| | Mean | 7·0 | 5·6 | 0·2 | 0·04 |
| | (st. error) | (0·2) | (0·4) | (0·03) | (0·01) |
| Resistant | 4 | 3·7 | 3·8 | — | — |
| | 8 | 4·3 | 3·6 | — | — |
| | 13 | 4·2 | 3·9 | — | — |
| | 28 | 5·0 | 3·5 | — | — |
| | 55 | 5·0 | 3·9 | — | — |
| | Mean | 4·4 | 3·7 | | |
| | (st. error) | (0·3) | (0·2) | | |
| MYLERAN | | | | | |
| Sensitive | 4 | 8·1 | 7·1 | 0·3 | 0·03 |
| | 8 | 7·6 | 6·3 | 0·4 | — |
| | 29 | 8·1 | 5·7 | 0·1 | 0·02 |
| | 52 | 10·0 | 5·6 | 0·1 | — |
| | 77 | 10·0 | 4·9 | 0·1 | 0·01 |
| | Mean | 8·7 | 5·9 | 0·2 | 0·02 |
| | (st. error) | (0·9) | (0·4) | (0·06) | (0·01) |
| Resistant | 4 | 2·7 | 3·3 | 0·2 | — |
| | 8 | 2·3 | 4·0 | — | — |
| | 29 | 3·8 | 3·3 | 0·2 | — |
| | 52 | 4·6 | 3·1 | — | — |
| | 77 | 4·1 | 3·4 | 0·3 | 0·05 |
| | Mean | 3·5 | 3·4 | 0·2 | — |
| | (st. error) | (0·4) | (0·2) | (0·04) | |

TABLE V.—*Subcellular Distribution of Glutathione in Sensitive Yoshida Ascites Sarcoma Cells from " Control " and Chlorambucil Treated (Curative Dose) Animals, Expressed in μmoles per 10⁹ Cells*

| GSH μmoles/10⁹ cells | Nuclear fraction | Mitochondrial fraction | Cell sap | Microsomal fraction |
|---|---|---|---|---|
| Sensitive controls | 0·5 | 0·3 | 4·9 | 0·1 |
| Sensitive treated | 0·7 | 0·5 | 19·6 | 0·1 |

However, at this time the sensitive cells were still 30 times more sensitive to melphalan than the resistant cells. At no time was GSSG detected, and the level of GSH in the resistant cells remained unaltered after drug treatment.

The RNA content of the sensitive cells increased by 36–67%, peak values occurring at 28 hours, 36 hours and 54 hours for Myleran, melphalan, and chlorambucil respectively (Fig. 9). No RNA changes were detected in the resistant cells.

The changes in protein content are shown in Fig. 10. Increases of 37–67% were observed in the sensitive cells, and the maxima were located at 28, 36 and 54 hours for Myleran, melphalan and chlorambucil respectively. No change was noted in the protein content of the resistant cells. No DNA changes were detected in either cell strain.

FIG. 8.—GSH content of Yoshida ascites sarcoma cells following " low dose " chemotherapy: ○, sensitive control; △, resistant control; ●——●——●, sensitive treated; ▲——▲——▲, resistant treated. The overall scatter about each point ≯10%. Each point represents the mean of three determinations.

FIG. 9.—RNA content of Yoshida ascites sarcoma cells following " low dose " chemotherapy: ○, sensitive control; △, resistant control; ●——●——●, sensitive treated; ▲——▲——▲, resistant treated. The overall scatter about each point ≯10%. Each point represents the mean of three determinations.

At this dose level the drugs elicited a maximum increase in cell volume of 50% (Harrap and Hill, 1969).

### DISCUSSION

### *Section I: " curative dose "*

This part of the study has revealed that the content of protein, RNA, glutamic acid, and glycine in the sensitive tumour cells was significantly higher than that of the resistant cells (before treatment with alkylating agents). The elevated amino acid levels do not imply necessarily a greater potenial for glutathione synthesis in the sensitive cells, since in this respect the concentration of cysteine would appear to represent the limiting substrate factor (Jackson, 1968): no cysteine was detected in either cell strain, and the levels of methionine and cystine were

FIG. 10.—Protein content of Yoshida ascites sarcoma cells following " low dose " chemotherapy: ○, sensitive control; △, resistant control; ●——●——●, sensitive treated; ▲——▲——▲, resistant treated. The overall scatter about each point ≯10%. Each point represents the mean of three determinations.

comparable. It is not known whether any particular component of the protein fraction was elevated in the sensitive cells, though other experiments have indicated that these contained twice as much γ-glutamylcysteine synthetase (EC 6.3.2.2.) as resistant cells (Harrap *et al.*, 1968).

Drug-induced increase in the nucleic acid, protein, and glutathione content of the sensitive cells occurred in two discrete phases: 0–24 hours, and post-24 hours respectively. These biochemical changes may be a counterpart of the altered growth-rate of the tumour cells resulting from treatment with the individual drugs.

During the 0–24-hour period following chlorambucil treatment the sensitive cells continued in logarithmic growth, while with Myleran the rate of proliferation decreased; on the other hand, treatment with melphalan resulted in the appearance of a plateau in the growth curve (Harrap and Hill, 1969). Hence, administration of Myleran and melphalan reduced the growth rate of sensitive cells during the 24 hours immediately following treatment, whereas the effects of chlorambucil were not apparent until 24 hours after the drug was given. This related behaviour of Myleran and melphalan, as distinct from chlorambucil, was endorsed by the accompanying biochemical changes: the pattern of nucleic acid, protein and glutathione increase in Myleran and melphalan-treated sensitive cells was broadly similar, but differed from that induced by chlorambucil (though certain quantitative differences from drug to drug were nevertheless detectable).

During the post-24 hour period, each drug induced comparable changes in the components studied. Extensive tumour cell death was occurring, and the biochemical changes observed must have represented the altered metabolism of lethally-damaged (yet living) cells. (It was important to recognise that drug-treated cells harvested throughout the course of these experiments were not dead cells—they continued to synthesise protein and nucleic acid—though they could not divide.) Other reports have suggested that DNA synthesis can be influenced by these agents without impairment of RNA or protein synthesis: the effects of alkylating agents on the content and synthesis of DNA in bacterial and mammalian cells have been reviewed recently by Ochoa and Hirschberg (1967).

The increases in glutathione levels which resulted from drug administration were at least two orders of magnitude greater, on a molar basis, than the amount of drug which could enter the cells (assuming a uniform distribution of the agent throughout the animal). Therefore, it seemed likely that the changes in glutathione content must have possessed a more sophisticated origin than replacement-synthesis of drug-alkylated thiol. The possibility that the increased level could be attributed to an accumulation of glutathione precursors has been eliminated in the present study. Furthermore, the appearance of oxidised glutathione in response to chlorambucil-treatment underlined the involvement of this drug with glutathione metabolism: the fact that peak levels occurred at different times (dependent on the cell strain) suggested that this drug may have selectively different effects on the glutathione oxido-reduction system of the cells. This possibility is being examined at the present time. In addition, the preliminary rise in glutathione content of the sensitive cells, which reached peak values at 12 hours after treatment with chlorambucil, may represent a selective mechanism for the inactivation of this drug (such an effect was not observed with Myleran or melphalan).

Drug treatment produced an increased protein content in the sensitive cells which paralleled a rise in cell volume (reported in the following paper (Harrap and Hill, 1969): presumably the dimensions of the cells increased in order to accommodate this elevated level of protein. However, the drug-induced increase in glutathione level could not be entirely associated with these events, for while the cell volume had doubled (at 60 hours) the glutathione content quadrupled.

The quantitative differences in response between sensitive and resistant cells could not be accounted for by selective differences in drug uptake: *in vitro* experiments have demonstrated that both cell strains accumulated comparable amounts of drug (Harrap and Hill, unpublished results).

The biological reactivity of these alkylating agents must be determined by competition between the rates of drug inactivation and DNA alkylation. It has been demonstrated that chlorambucil could mobilise a nucleophilic cell constituent (glutathione) before the effects of DNA alkylation had been observed, thus increasing the number of alkylating sites available to the drug. Myleran and melphalan could not mobilise GSH within this 24-hour period. These findings imply variations in the mechanism of detoxication of the drugs. Such differences may appear surprising in view of the similarities in structure and half-life of hydrolysis of chlorambucil and melphalan. However, *in vitro* experiments have demonstrated that the alkylating abilities of these two drugs differ markedly once they have been absorbed by the cells (Harrap and Hill, unpublished results). This suggests that in addition to loss of drug by alkylating side reactions, these agents may also be sequestered in an active form, possibly by protein. This speculation is supported by the observations of Wade *et al.* (1967).

The metabolic response of the cells to chlorambucil was clearly faster than to Myleran and melphalan (the reverse of the biological response) and it becomes important to determine the rate at which these drugs selectively alkylate DNA in order to assess the relative importance of the biochemical events described here.

*Section II: " therapeutically ineffective dose "*

It could be argued that the metabolic alterations discussed above were impressed on the cells by alkylated DNA, and that the changes reflected the altered function of lethally damaged genetic material. It was necessary to investigate this possibility, and to examine to what extent the changes were representative of the interaction of the alkylating agents with cellular components. This objective was achieved by examining the alterations produced by " therapeutically ineffective " doses of the drugs, from which the sensitive cells would recover. Under this circumstance the observed biochemical changes could not be ascribed to metabolic irregularities arising from the lethal alkylation of DNA.

A reversible accumulation of RNA, protein and GSH occurred in the sensitive cells, though the DNA content remained unaltered: no changes were observed in the levels of these compounds in the resistant cells. These effects should be compared with the irreversible accumulation of DNA, RNA, protein, and GSH in sensitive cells following the administration of " curative " doses of the agents.

Again the cell volume increased presumably in order to accommodate the raised level of RNA and protein. The accompanying rise in GSH, as with curative drug doses occurred in greater proportion to the cell volume increase, and probably was not associated with the increase in protein and RNA content. This point may be amplified by reference to Table VI: fractionated doses of melphalan

TABLE VI.—*Effect of " Fractionated " Doses of Melphalan on GSH, RNA, Protein Content of Sensitive Yoshida Ascites Cells*

| Dose (mg./kg.) | GSH (μmoles/10⁹ cells) | increase (%) | RNA (mg./10⁹ cells) | increase (%) | Protein (mg./10⁹ cells) | increase (%) |
|---|---|---|---|---|---|---|
| 0 | 6 | | 39 | | 120 | |
| 0·016 | 8·5 | 42 | 46·0 | 18 | 152 | 27 |
| 0·16 | 13·2 | 112 | 48·7 | 25 | 177 | 47 |

produced much larger effects on the GSH content of sensitive cells than on their protein or RNA contents. If the effects elicited by a dose of 0·16 mg./kg. were related to a baseline of 0·016 mg./kg., then for this 10-fold increase in dose, the rise in GSH was approximately four times the protein, and nine times the RNA elevation.

The evidence discussed in the two sections above implies that these drugs disturb the metabolism of several cellular constituents, namely:

(i) DNA
(ii) RNA and protein
(iii) GSH.

(i) Curative doses of drug resulted in an accumulation of DNA, while the DNA content remained unchanged following low (therapeutically ineffective) doses. (ii) An irreversible accumulation of RNA and protein occurred (paralleled by a corresponding change in cell volume) following curative doses, while the low doses yielded reversible increases in these components (again with a corresponding change in cell volume). (iii) Drug-induced increase in glutathione content was always considerably greater than the changes observed in any other component at either dose level.

Although broad similarities were detected in the pattern of response to ineffective doses of each drug, the reversible changes observed were separable from drug to drug in terms of their time and duration of onset, indicating a measure of individuality of action. It is interesting that the order of reactivity of these drugs, as measured here by the time of peak accumulation of the various components, correlated well with their effectiveness (at " curative dose " levels) on the tumour growth rate (Harrap and Hill, 1969), and again was the inverse of the chemical reactivities of the agents.

### SUMMARY

A strain of the Yoshida ascites sarcoma which responded to chemotherapy with alkylating agents contained elevated levels of RNA, protein, glutamic acid and glycine, when compared with a drug-resistant strain. Curative treatment with individual alkylating agents resulted in the accumulation of DNA, RNA, protein and GSH in the sensitive cells. These reactions occurred during two time intervals: 0–24 hours and post-24 hours. Events occurring during the second period were broadly similar, irrespective of the identity of the drug used, while in the first period each drug elicited qualitatively and quantitatively different effects. In all cases the relative increase in glutathione content was greater than that of the other components measured.

A reversible accumulation of glutathione, RNA and protein occurred in the sensitive cells following the administration, to the host rats, of therapeutically ineffective doses of a number of alkylating drugs. The time course of these changes differed according to the identity of the drug used, and provided a measure of the selectively different action of the latter on the cells. Similar effects were not observed in a resistant strain of cells.

It is proposed that alkylating agents may have independent effects on several cellular constituents: DNA, RNA and protein; glutathione.

The authors are indebted to Dr. T. A. Connors and Dr. C. Ball for the supply of tumour-bearing animals, and for much helpful advice and discussion. They are also grateful to Miss C. A. Smith for skilled technical assistance. Thanks are also due to Dr. D. F. Evered (Chelsea College of Science and Technology) who provided the facilities for high voltage electrophoresis and automatic amino acid analyses. One of us (B.T.H.) acknowledges the receipt of an S.R.C. maintenance grant. This investigation has been supported by grants to the Chester Beatty Research Institute (Institute of Cancer Research: Royal Cancer Hospital) from the Medical Research Council and the British Empire Cancer Campaign for Research, and by Public Health Service Research Grant No. CA-03188-10 from the National Cancer Institute, U.S. Public Health Service.

### REFERENCES

Ball, C. R., Connors, T. A., Double, J. A., Ujhazy, V. and Whisson, M. E.—(1966) *Int. J. Cancer*, **1**, 319.

Boesen, E., Galton, D. A. G. and Wiltshaw, E.—(1964) In ' Chemotherapy of Cancer ', edited by Plattner. London (Elsevier), 51–61.

BOULTER, D.—(1966) ' An introduction to automatic amino acid analysis with plant extracts '. Technicon Monograph No. 1, p. 93.
BROOKES, P. AND LAWLEY, P. D.—(1961) *Biochem. J.*, **80**, 496.
BROWN, A. M.—(1946) *Archs Biochem.*, **11**, 269.
BURTON, K.—(1956) *Biochem. J.*, **62**, 315.
CALCUTT, G. AND CONNORS, T. A.—(1963) *Biochem. Pharmac.*, **12**, 833.
CONNORS, T. A., DOUBLE, J. A., ELSON, L. A. AND JENEY, A., JR.—(1965) *Biochem. Pharmac.*, **14**, 569.
CRATHORN, A. R. AND ROBERTS, J. J.—(1966) *Nature, Lond.*, **211**, 150.
DOHAN, J. S. AND WOODWARD, G. E.—(1939) *J. biol. Chem.*, **129**, 393.
ELLMAN, G. L.—(1959) *Archs Biochem. Biophys.*, **80**, 70.
HAMILTON, P. B.—(1963) *Analyt. Chem.*, **35**, 2055.
HARRAP, K. R.—(1967) *Biochem. Pharmac.*, **16**, 725.
HARRAP, K. R. AND HILL, B. T.—(1969) *Br. J. Cancer*, **23**, 227.
HARRAP, K. R., JACKSON, R. C. AND HILL, B. T.—(1968) 5th Meeting of the Federation of European Biochemical Societies, Prague, Abstr. No. 299.
HARRAP, K. R. AND SPEED, D. E. M.—(1964) *Br. J. Cancer*, **18**, 809.
HIRONO, I.—(1961) *Gann*, **52**, 39.
HIRONO, I., KACHI, H. AND OHASHI, A.—(1962) *Gann*, **53**, 73.
JACKSON, R. C.—(1968) *Biochem. J.*, **111**, 301.
KOHN, K. W., STEIGBIGEL, N. H. AND SPEARS, C. L.—(1965) *Proc. natn. Acad. Sci. U.S.A.*, **53**, 1154.
KUN, E., LANGER, B., ULRICH, B., HOLZER, H. AND GRUNICKE, H.—(1964) *Proc. natn. Acad. Sci. U.S.A.*, **52**, 1501.
LAWLEY, P. D. AND BROOKES, P.—(1965) *Nature, Lond.*, **206**, 480.
LOVELESS, A.—(1966) ' Genetic and allied effects of alkylating agents '. London (Butterworth).
LOWRY, O., ROSEBROUGH, N. J., FARR, A. L. AND RANDALL, J.—(1951) *J. biol. Chem.*, **193**, 265.
Medical Research Council's working party of therapeutic trials in leukaemia.—(1968) *Br. med. J.*, i, 201.
OCHOA, Jr., M. AND HIRCHBERG, E.—(1967) *Expl. Chemother.*, **5**, 1.
RUNDLES, R. W. (1967) In: ' Cancer Chemotherapy ', 15th Hahnemann Symposium, edited by Brodsky and Kahn. pp. 229–238.
SCHMIDT, D.—(1966) ' Techniques in amino-acid analysis '. Technicon Monograph No. 1.
SMITH, I.—(1958) ' Chromatographic techniques '. London (Heinemann).
SPACKMAN, D. A., STEIN, W. H. AND MOORE, S.—(1958) *Analyt. Chem.*, **30**, 1190.
THERKELSEN, A. J.—(1958) *Biochem. Pharmac.*, **1**, 258.—(1961) *Biochem. Pharmac.*, **8**, 269.
UJHAZY, V. AND WINKLER, A.—(1965) *Neoplasma*, **12**, 11.
VOLKIN, E. AND COHN, W. E.—(1954) *Meth. biochem. Analysis*, **1**, 287.
WADE, R., WHISSON, M. E. AND SZEKERKE, M.—(1967) *Nature, Lond.*, **215**, 1303.
WALTON, R. L., TAYLOR PETERSON, E. AND DOYLE, P.—(1957) *Blood*, **12**, 953.
WARWICK, G. P.—(1963) *Cancer Res.*, **23**, 1315.